# *Hsa-miR-155-5p* drives aneuploidy at early stages of cellular transformation

**DOI:** 10.18632/oncotarget.24437

**Published:** 2018-02-07

**Authors:** Sara Pagotto, Angelo Veronese, Alessandra Soranno, Paola Lanuti, Mirco Di Marco, Marco Vincenzo Russo, Alice Ramassone, Marco Marchisio, Pasquale Simeone, Paolo E. Guanciali Franchi, Giandomenico Palka, Renato Mariani Costantini, Carlo M. Croce, Rosa Visone

**Affiliations:** ^1^ Aging Research Center and Translational Medicine-CeSI-MeT, Chieti, 66100, Italy; ^2^ Department of Medical, Oral and Biotechnological Sciences, “G. d'Annunzio” University Chieti-Pescara, Chieti, 66100, Italy; ^3^ Department of Medicine and Aging Sciences, “G. d’Annunzio” University Chieti-Pescara, Chieti, 66100, Italy; ^4^ Department of Molecular Virology, Immunology, and Medical Genetics, Comprehensive Cancer Center, The Ohio State University, Columbus, Ohio 43210, USA; ^5^ Chronic Lymphocytic Leukemia Research Consortium, San Diego, California 92093, USA

**Keywords:** miR-155, BUB1, CENP-F, ZW10, aneuploidy

## Abstract

*Hsa-miR-155-5p (miR-155)* is overexpressed in most solid and hematological malignancies. It promotes loss of genomic integrity in cancer cells by targeting genes involved in microsatellite instability and DNA repair; however, the link between *miR-155* and aneuploidy has been scarcely investigated. Here we describe a novel mechanism by which *miR-155* causes chromosomal instability. Using osteosarcoma cells (U2OS) and normal human dermal fibroblast (HDF), two well-established models for the study of chromosome congression, we demonstrate that *miR-155* targets the spindle checkpoint proteins BUB1, CENP-F, and ZW10, thus compromising chromosome alignment at the metaphase plate. In U2OS cells, exogenous *miR-155* expression reduced the recruitment of BUB1, CENP-F, and ZW10 to the kinetochores which resulted in defective chromosome congression. In contrast, during *in vitro* transformation of HDF by enforced expression of SV40 Large T antigen and human telomerase (HDF_LT/hTERT_), inhibition of *miR-155* reduced chromosome congression errors and aneuploidy at early passages. Using live-cell imaging we observed that *miR-155* delays progression through mitosis, indicating an activated mitotic spindle checkpoint, which likely fails to reduce aneuploidy. Overall, this study provides insight into a mechanism that generates aneuploidy at early stages of cellular transformation, pointing to a role for *miR-155* in chromosomal instability at tumor onset.

## INTRODUCTION

*Hsa-miR-155-5p* (*miR-155*) is one of the first oncogenic miRNAs described in leukemias, breast, lung and colon cancers [1–3]. Several oncogenic roles have been ascribed to this *miR*, including a contribution to genomic instability through the down-regulation of transcripts implicated in DNA mismatch repair, DNA damage repair, and maintenance of telomere integrity [4–6]. However, genomic instability can also be due to chromosome instability (CIN), which has important implications for tumor initiation [7, 8] and evolution [9, 10].

CIN arises mostly through defects in the spindle assembly checkpoint (SAC), a quality control mechanism that inhibits anaphase onset until all chromosomes are correctly attached to the spindle microtubules via the kinetochores [11–13] and is rarely caused by telomere erosion [14–16]. The SAC depends on proteins of the outer plate of the kinetochore, such as the centromere protein F (CENP-F), the zw10 kinetochore protein (ZW10) and the mitotic checkpoint serine/threonine kinase budding uninhibited by benzimidazoles 1 (BUB1), one of the most important SAC kinases [17]. Loss of these proteins severely affects chromosomal segregation at anaphase, leading to aberrant aneuploid cells that are thought to be selected for proliferative and adaptive advantages linked with transformation [17–20].

Given that CIN is a cancer hallmark and that *miR-155*, involved in several tumors, affects molecular pathways involved in genomic stability, we hypothesized that this miRNA could interfere also with the molecular complexes of the SAC, leading to aneuploidy in cancer cells.

To demonstrate this, we investigated whether *miR-155* could: i) directly influence the expression of the SAC complex proteins BUB1, ZW10 and CENP-F; ii) trigger chromosome alignment defects during cellular transformation; iii) prolong mitosis and increase the rate of aneuploidy at early stages of HDF immortalization. We found that *miR-155,* acting through the SAC, contributed to generate aneuploidy during cellular transformation.

## RESULTS

### *MiR-155* negatively regulates BUB1 protein expression

**Figure 1:**
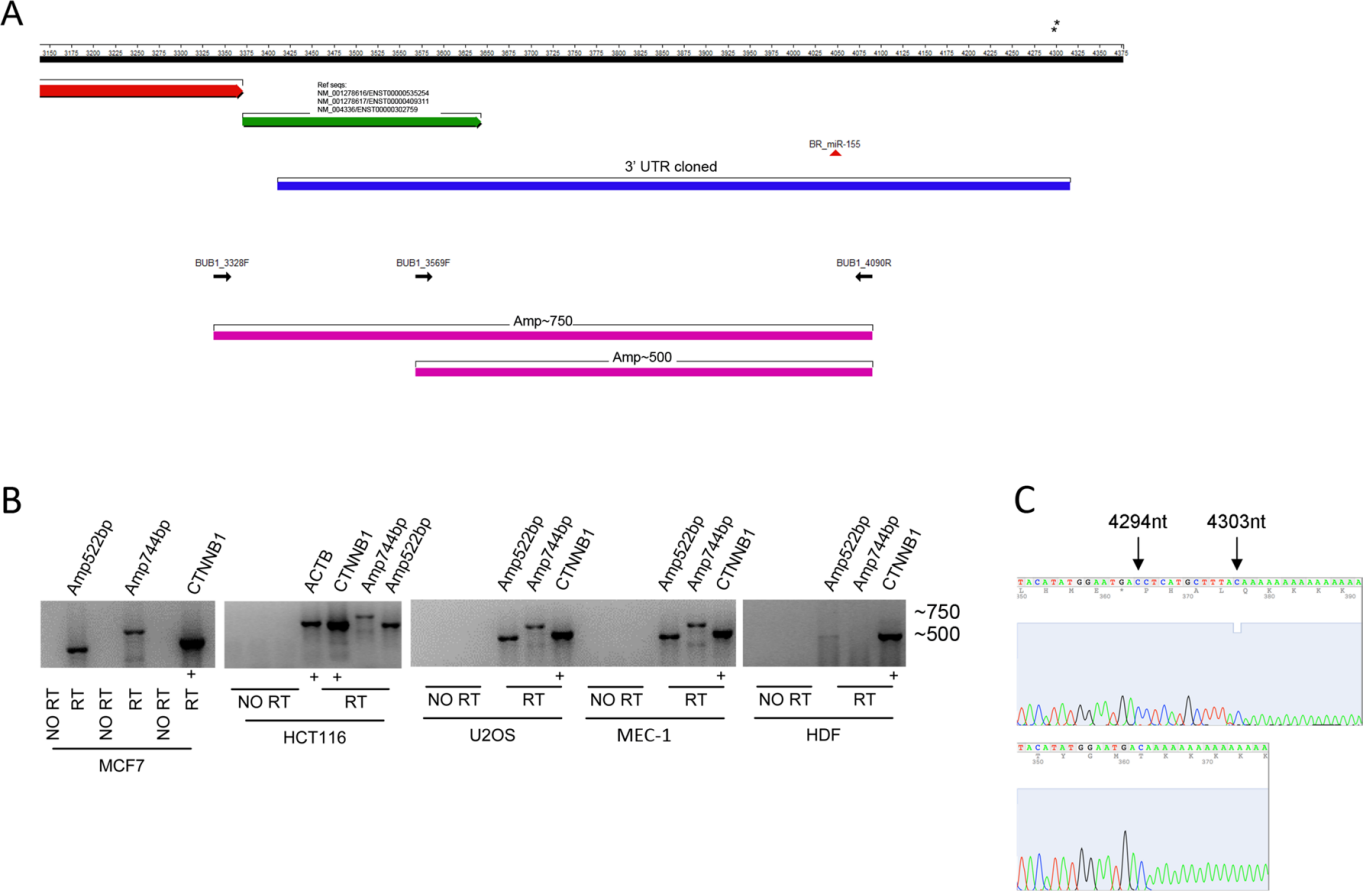
BUB1 3’UTR analysis (**A**) Schematic representation of the 3’UTR of the *BUB1* gene shows, from top to bottom: nucleotide numbering (RefSeq NM_004336; black bar). Black asterisks indicate the 3′ends of the 3’UTR determined by 3′RACE; 3′end of the coding sequence (red arrow); NCBI/Ensembl reference 3’UTR sequence (NCBI/Ensemble; green arrow); 3’UTR (blue bar) as cloned into psiCHECK-2 reporter vector; predicted miR-155 binding site (red triangle); and PCR primers (black arrows) marking two amplicons of ~500 bp and ~750 bp (magenta bars). (**B**) Detection of the long mRNA isoform of *BUB1* in four human cancer cell lines and normal human dermal fibroblasts (HDF) by RT-PCR followed by gel electrophoresis (1.5% agarose gel in 1X TBE buffer) and ethidium bromide staining. *No RT*, negative control without reverse transcriptase. CTNNB1 and ACTB, internal positive controls with similar fragment size to the BUB1 amplicons. (**C**) Sequence chromatograms of two different products obtained by the 3′rapid amplification cDNA ends (RACE) workup of HG-3 cell RNA to determine the 3′end sequence of *BUB1* 3’UTR. Blacks asterisks in panel A mark the 3′ends of the cloned 3′UTR. The specific primers used are listed in Supplementary Table 2.

**Figure 2:**
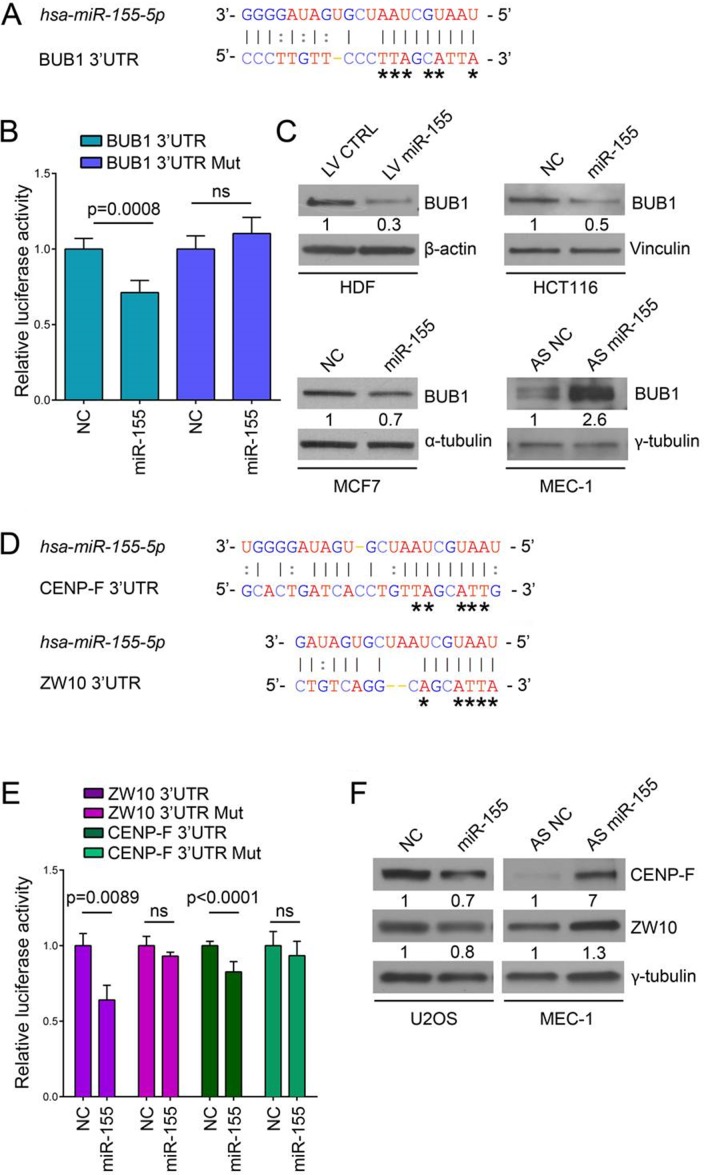
BUB1, CENP-F and ZW10 are targeted by *miR-155* (**A**) Base pairing between *miR-155* (top) and predicted binding site in *BUB1* 3’UTR (bottom) according to MicroCosm Targets version 5). Asterisks indicate nucleotides subject to site-directed mutagenesis. Watson-Crick base pairing (G-C and A-T) is marked by vertical lines while G-T pairs are marked by two dots. (**B**) Relative luciferase activity in U2OS cells transfected with psiCHECK-2_BUB1 3’UTR with either the predicted miR-155 binding site in reference sequence or mutated (Mut) form and either the miR-155 precursor (miR-155) or the negative control oligonucleotide (NC). Values are means ± SD of five technical replicates, ns: no significant *p*-value. (**C**) Western blots of BUB1 protein (120 kDa) after miR-155 lentiviral infection in HDF cells and after transfection with miR-155 precursor, antisense inhibitor oligonucleotide (AS miR-155), or respective negative controls (NC and AS NC) in HCT116, MCF7 and MEC-1 cell lines. (**D**) Base pairing between *miR-155* and predicted binding sites in *CENP-F* 3’UTR and *ZW10* 3’UTR, according to MicroCosm Targets version 5. Asterisks indicate nucleotides subject to site-directed mutagenesis. (**E**) Relative luciferase activity in U2OS cells transfected with psiCHECK-2_CENP-F 3’UTR or psiCHECK-2_ZW10 3’UTR with the predicted miR-155 binding site in reference or mutated form, and either miR-155 or NC. Values are means ± SD of four technical replicates for ZW10 and eight measures from two independent experiments for CENP-F (4 technical replicates from each experiment). (**F**) Western blots of CENP-F and ZW10 proteins after transfection with miR-155 precursor, antisense of miR-155 or respective negative controls (NC and AS NC) in U2OS and MEC-1 cells as indicated.

To investigate a possible link between *miR-155* and CIN we looked for new putative targets of *miR-155*. *In silico* analyses provided by the MicroCosm Targets Version 5 database revealed that BUB1 is a predicted target of *miR-155*. However, this database refers to a discontinued *BUB1* sequence (ENST00000389944, Ensembl) with a 3’UTR more extended than the current reference sequences (transcript variant 1: NM_004336/ENST00000302759; transcript variant 2: NM_001278616/ENST00000535254; transcript variant 3: NM_001278617/ENST00000409311; NCBI/Ensembl), that exclude the predicted *miR-155* binding site (Figure 1A). We verified whether the *BUB1* transcript with the extended 3’UTR was naturally expressed in normal and cancer cells. For this purpose we amplified by RT-PCR two different fragments (Amp~500; Amp~750) (Figure 1A) of the putative 3’UTR from HDF, and from a panel of cancer cells representative of tumors of breast (MCF7), colon (HCT116), of chronic lymphocytic leukemia (CLL: MEC-1), and osteosarcoma (U2OS), neoplasms for which the relevance of *miR-155* has been well demonstrated [1–3, 21, 22]. Gel electrophoresis of the RT-PCR products showed the expected ~500 bp fragments in all the analyzed cell types, and the ~750 bp fragments in all the cancer cell lines, confirming the presence of the long transcript isoform (Figure 1B). In HDF cells we detected only the ~500 bp fragment, the ~750 bp fragments was undetectable, which is probably due to the technical issues related to the low expression of the gene in cells replicating slowly. To characterize the 3′end of the long isoform, we performed 3′ Rapid Amplification cDNA Ends (RACE) using RNA from the HG-3 B-CLL cell line. We identified two close 3’UTR ending points at positions 4294 and 4303 of the *BUB1* sequence that includes the miR-155 binding region (Figure 1C). Next, we validated *BUB1* as a direct target of *miR-155* by luciferase assay in U2OS cells. Compared to the respective controls, *miR-155* suppressed the luciferase activity of the construct containing the native *BUB1* 3’UTR by ~30%, while the luciferase activity of the construct containing the mutated *BUB1* 3’UTR was unaffected. This demonstrates that *miR-155* directly interacts with the 3’UTR of *BUB1* (Figure 2A and 2B).

To further assess the influence of *miR-155* on BUB1 expression, we evaluated changes in BUB1 protein levels by western blotting of HDF infected with a lentiviral expression vector carrying *miR-155* (LV miR-155) or a control vector (LV CTRL). Overexpression of *miR-155* strongly decreased the BUB1 protein levels (–70%). Concordantly, the BUB1 levels decreased after ectopic *miR-155* expression in MCF7 (–30%) and HCT116 (–50%) cells. Furthermore, BUB1 protein expression strongly increased when MEC-1 cells, which present high levels of endogenous *miR-155*, were transfected with an antisense miR-155 (Figure 2C, Supplementary Figure 1). Overall, these results indicate that *miR-155* binds to the 3’UTR of *BUB1* and specifically represses BUB1 protein expression.

### ZW10 and CENP-F are direct targets of *miR-155*

To strengthen the relevance of the *miR-155* in the mitotic spindle assembly we looked for additional targets among kinetochore proteins and found two additional SAC proteins: ZW10 and CENP-F. (MicroCosm Targets Version 5 database) (Figure 2D). Direct targeting by *miR-155* was demonstrated using a luciferase assay, as before. The luciferase activity of constructs containing *ZW10* and *CENP-F* 3′UTRs decreased by ~40% and ~20%, respectively after ectopic expression of *miR-155*, while these effects were abolished when the 3’UTRs were altered by mutagenesis (Figure 2E). Western blotting showed that induced expression of *miR-155* in U2OS cells decreased ZW10 (-20%) and CENP-F (-30%) protein levels, while in MEC-1 cells ZW10 and CENP-F protein expressions increased after modulation of *miR-155* with an antisense *miR-155* (Figure 2F). Collectively, these results demonstrate that *miR-155* directly regulates ZW10 and CENP-F protein levels, which supports a role of this miRNA in the regulation of mitotic progression trough the SAC.

### *MiR-155* affects chromosome congression and outer plate kinetochore assembly

**Figure 3:**
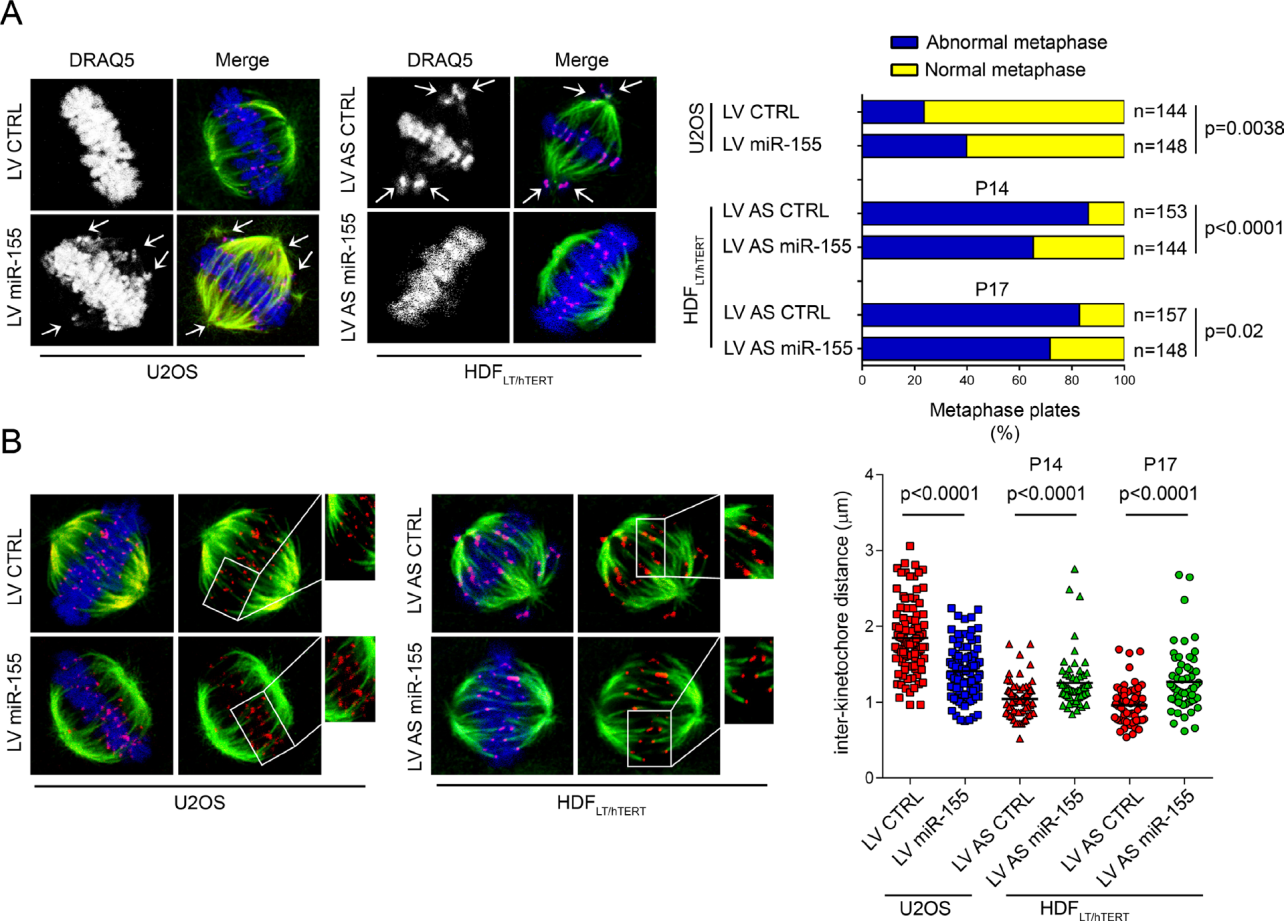
Role of *miR-155* in chromosome congression (**A**) Left, Immunofluorescence of HDF_LT/hTERT_ and U2OS cells stained for α-tubulin (green), α-centromere (red), and DNA with DRAQ5 (blue). Both cell types, infected as indicated, arrested at the metaphase-anaphase transition upon treatment with the proteasome inhibitor MG132 (U2OS, 10 μM for 2 h) or nocodazole (HDF_LT/hTERT_, 75 ng/ml for 17 h plus 30 min of release). Chromosomes in metaphase cells were counted as unaligned if they were located outside of the mitotic spindle or if their kinetochores were aligned perpendicularly to the spindle axis (white arrows). Right, Quantification of U2OS and HDF_LT/hTERT_ congression errors in two independent experiments at P14 (Experiment 1) and P17 (Experiment 2). (**B**) Inter-kinetochore distances measured in prometaphase-metaphase of cells infected as indicated and stained with anti-α-tubulin (green) or anti-centromere (red) antibodies and DRAQ5 (blue). Over 60 kinetochore pairs in more than 10 cells were examined for each condition.

Chromatids of cells depleted of BUB1 do not align correctly on the spindle equator before anaphase [17], ZW10-deficient cells show aberrant mitoses and abnormal chromosome distribution as well, with formation of chromatin bridges and micronuclei [18] and CENP-F repression induces defects in chromosome alignment [19, 20]. Therefore, we hypothesized that *miR-155* could affect the accuracy of mitotic chromosome congression by targeting these kinetochore proteins. To test this hypothesis, we analyzed the morphology of the metaphase plates after induction of *miR-155* in U2OS cells infected with LV miR-155 (Supplementary Figure 2A), and found that the percentage of irregular metaphases was higher (~40%) than in control cells infected with LV CTRL (~24%; *p* = 0.0038) (Figure 3A, right). To further evaluate this mechanism, we transformed normal HDF by sequential infection with retroviruses that transduce first the simian virus 40 (SV40) large T antigen (LT) and then the catalytic subunit of human telomerase (hTERT) (HDF_LT/hTERT_) [23, 24]. We decided to use this *in vitro* model because of 1) the possibility to analyze metaphases during the cellular transition from normal to abnormal karyotypes and 2) the possibility to modulate *miR-155* expression, since its expression increased starting from the very early passage of infection in HDF_LT/hTERT_ cells (Supplementary Figure 2B). We analyzed the morphology of the metaphase plates in HDF_LT/hTERT_ cells infected with either LV AS miR-155 or LV AS CTRL at low passages (P14 and P17). Metaphases with mal-oriented chromosomes represented ~86% (P14) and ~83% (P17) of the total in HDF_LT/hTERT_ controls, and ~65% (P14) and ~72% (P17) of the total in LV AS miR-155 HDF_LT/hTERT_ cells (P14, *p* < 0.0001; P17, *p* = 0.02) (Figure 3A, right). Regulation of BUB1, CENP-F and ZW10 by LV AS miR-155 in HDF_LT/hTERT_ and LV miR-155 in U2OS cells is shown in Supplementary Figure 3.

**Figure 4:**
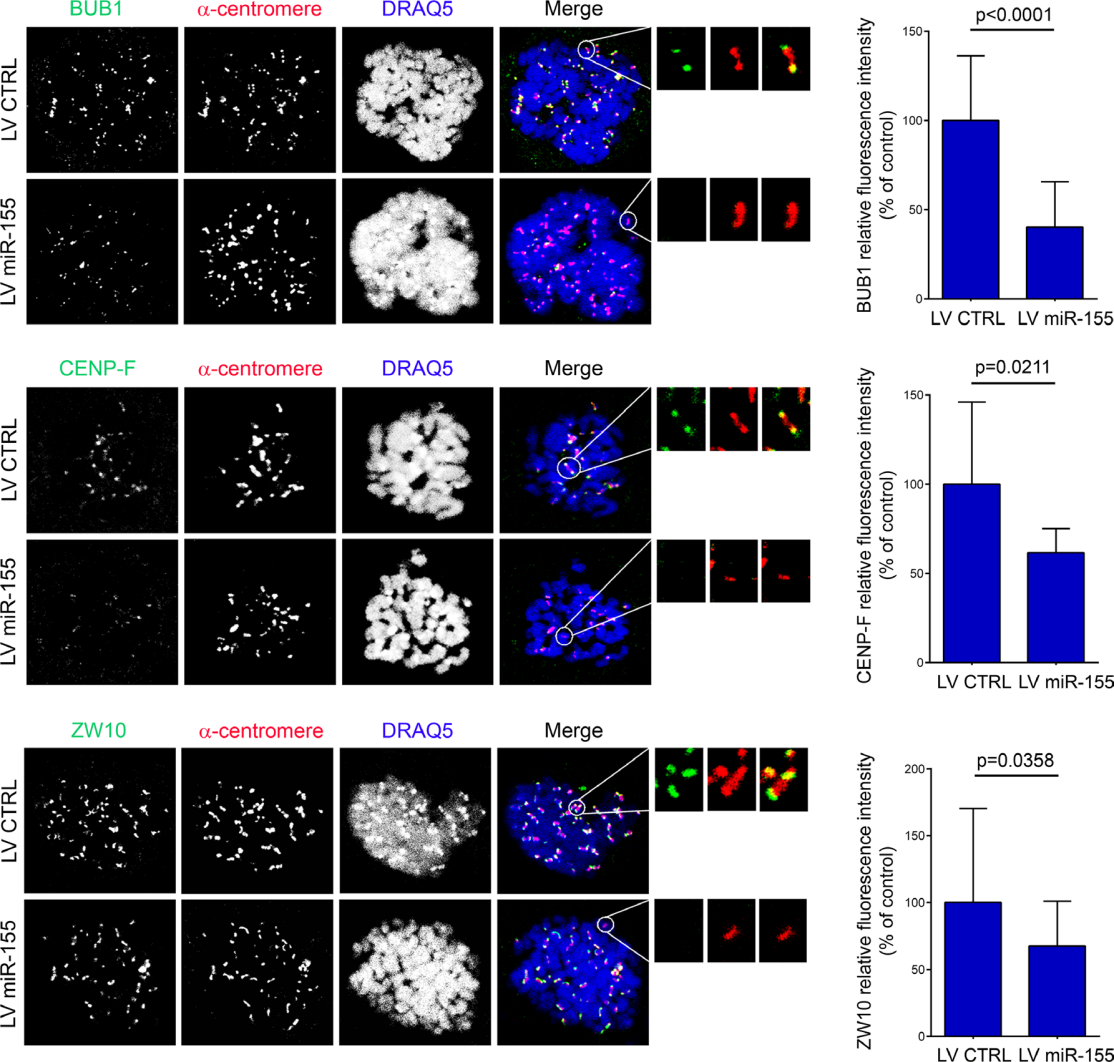
Over-expression of *miR-155* affects the recruitment of BUB1, CENP-F and ZW10 U2OS cells infected with LV miR-155 and control were arrested at prometaphase by nocodazole treatment and then fixed and labeled with the indicated antibodies. Enlargements of kinetochores are circled in the merged image and shown in the inserts. Graphs present the fluorescence intensity of the protein normalized to that of anti-centromere staining. The bar graphs were derived from measurements of all the kinetochores in at least 10 cells.

Tension within the kinetochore generated by the microtubules attached to the opposite spindle poles is crucial for proper chromosome configuration [25–27]. To further explain the errors in chromosome alignment associated with the deregulation of *miR-155*, we measured the inter-kinetochore distances as indirect indicators of tension in the centromere-kinetochore region of the sister chromatids. The inter-kinetochore distances of metaphase chromosomes significantly increased in HDF_LT/hTERT_ infected with LV AS miR-155. On the other hand, in the U2OS cells over-expressing the *miR*, the distances between kinetochores were significantly reduced (Figure 3B). Next, we examined by immunofluorescence whether overexpression of *miR-155* reduced the recruitment of BUB1, CENP-F and ZW10 at the kinetochores. In LV miR-155-infected U2OS cells the BUB1, CENP-F and ZW10 immunofluorescence intensity levels were reduced by – 60% (*p* < 0.0001), – 38% (*p* = 0.0211), and – 32% (*p* = 0.0358), respectively (Figure 4).

### *MiR-155* is required to block cell cycle progression at the G2/M phase and to delay mitosis exit

**Figure 5:**
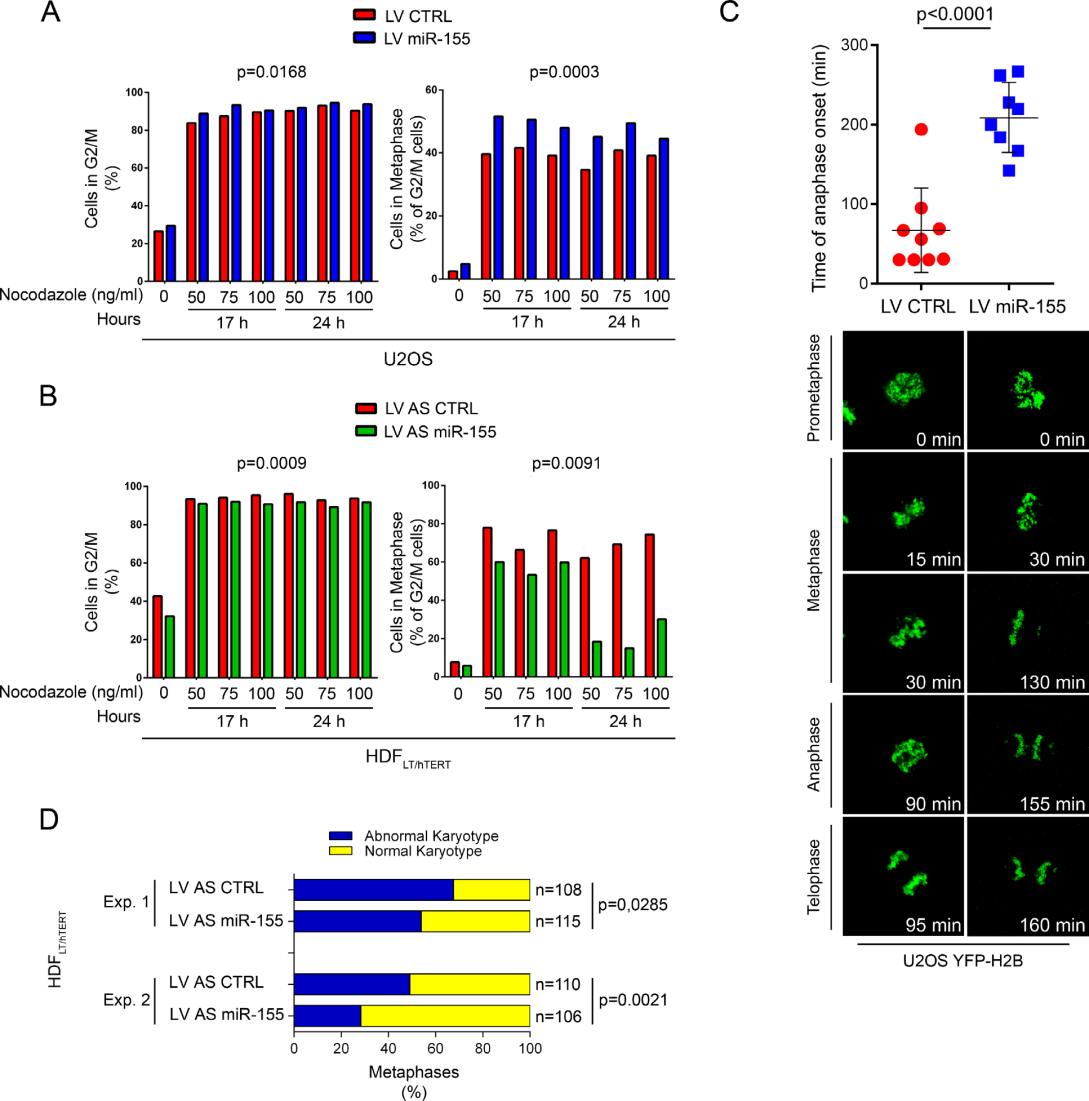
Over-expression of *miR-155* blocks cells at G2/M phase and prolongs the duration of metaphase by delaying chromosome congression Flow citometry of (**A**) U2OS cells infected with LV miR-155 and LV CTRL and (**B**) HDF_LT/hTERT_ infected with LV AS miR-155 and LV AS CTRL. As indicated, cells were blocked at different concentrations of nocodazole and for different times. Left, G2/M cells were determined by quantifying nuclear DNA and Right, mitotic cells by anti-phospho-histone-H3 (PH-H3) staining gated on G2/M cells. (**C**) Time of anaphase onset in U2OS cells stably expressing YFP-H2B and infected with the indicated lentivirus. Time lapse measuring started at prometaphase (*T* = 0), and images were acquired every 5 min. Individual measurements (dots) with mean and standard deviations are represented in the graph. (**D**) Karyotype analysis of two independent experiments (Experiment 1, Experiment 2) at P14. HDF_LT/hTERT_ cells were arrested in mitosis by colcemid treatment, harvested for chromosome spread and then stained with Giemsa solution. A minimum of 100 metaphases was considered in each experiment.

Defects in metaphase plate alignment influence cell cycle progression. Thus, using in U2OS cells, we evaluated the effects of *miR-155* on the cell cycle after reversible damage induced by nocodazole. The percentage of miR-155-infected U2OS cells in G2/M was significantly higher than in control U2OS cells (*p* = 0.0168), and this corresponded to an increased mitotic index (*p* = 0.0003) (Figure 5A). This finding suggests that cells with high *miR-155* expression delay their exit from G2/M after nocodazole-induced damage. We corroborated our results by replicating this experiment in HDF_LT/hTERT_ with or without LV AS miR-155. Compared to controls, the fraction of miR-155-depleted HDF_LT/hTERT_ cells blocked in G2/M was significantly lower (*p* = 0.0009), which corresponded to a reduction in mitotic index of the metaphases (*p* = 0.0091) (Figure 5B).

The effect of *miR-155* during mitosis was also evaluated using live-cell imaging technology. To analyze chromosome dynamics, we generated U2OS cells stably expressing the yellow fluorescent protein-histone H2B fusion protein (YFP-H2B) and either *miR-155* (by infection with LV miR-155) or a control (by infection with LV CTRL). In U2OS cells infected with LV CTRL mitosis proceeded from prometaphase to anaphase in a mean of 68 minutes, with chromosomes perfectly aligned on the metaphase plate (Supplementary Movie 1). In U2OS cells overexpressing *miR-155* (LV miR-155), progression from prometaphase to anaphase took longer (mean, 209 min) (Supplementary Movie 2), chromosomes failed to align to the metaphase plate and, after a long delay, the cells frequently exited mitosis without clear anaphase and with lagging chromatids (Figure 5C). These results demonstrate a strong activation of the SAC in cells with chromosomal congression defects, even though at the end most of the cells completed cell division (data not shown).

### Over-expression of *miR-155* promotes aneuploidy at the onset of transformation

*BUB1* hypomorphic mice have a high percentage of aneuploid cells and are predisposed to spontaneous tumors [28, 29]. CENP-F expression is associated with poor prognosis and chromosomal instability in primary breast cancer [30], and mutational screening in human colorectal cancers has identified mutations in *ZW10*, that may contribute to CIN [31]. To determine if *miR-155* causes aneuploidy, we analyzed the karyotypes of HDF_LT/hTERT_ cells infected with either LV AS miR-155 or LV AS CTRL at an early passage (P14). Since CIN can affect both entire chromosomes and parts of them, we counted loss and gain of whole chromosomes as well as chromosomal amplifications, unbalanced translocations, and deletions. Transformed HDF cells in which *miR-155* was inhibited(HDF_LT/hTERT_ LV AS miR-155) maintained a near-diploid karyotype, while control cells had marked aneuploidy and chromosomal instability (Experiment 1, *p* = 0.0285; Experiment 2, *p* = 0.0021) (Figure 5D, Supplementary Table 1).These results strengthen the direct link between *miR-155* and CIN/aneuploidy.

## DISCUSSION

This study reveals a novel link between *miR-155* and aneuploidy. We demonstrated that *miR-155* directly targets three key proteins of the mitotic SAC, namely BUB1, CENP-F and ZW10 [32–36]. Enhanced expression of *miR-155* in our cellular models caused defects in chromosome alignment associated with altered distances between sister kinetochores and a moderate-to-severe decrease of these proteins levels to the kinetochores after nocodazole-induced damage. Given the abundantly demonstrated role of BUB1, CENP-F and ZW10 in inducing defects of the mitotic spindle after their depletion, we strongly suggest that the mechanism by which *miR-155* induces metaphase plate misalignments and in turn aneuploidy is through the direct down-regulation of these proteins. Moreover, this also reflects an important role of *miR-155* in the duration of the cell division. Consistently, *miR-155* blocked nocodazole-treated cells in metaphase and increased their mitotic index. Indeed, delayed mitosis is the proper reaction of a cell to misaligned chromosomes, as it enables the correction of errors but, in a pre-cancerous context that could involve chronic inflammation and/or viral infection and/or genetic predisposition, cells with up-regulation of *miR-155* may acquire an anti-apoptotic support to exit from the G2/M phase, despite chromosome alignment defects at the metaphase plate.

It is commonly accepted that tumor progression is fueled by a gradual accumulation of genetic anomalies, and that higher genetic instability correlates with more aggressive and metastatic forms of disease [9, 37]. However, recent studies showed that aneuploidy occurs early in carcinogenesis followed by a period of genomic stability [38–42]. Accordingly, we confirmed that *miR-155* is involved in CIN at an early stage of transformation (14th passage), since in our *in vitro* model, inhibition of *miR-155* decreased the number of cells with abnormal karyotypes after infection with transforming viruses. We took advantage of the ability of the SV40 large T antigen to generate aneuploidy [43], which is the resultant of its direct interaction with the tumor suppressor protein TP53 [44, 45], with members of the retinoblastoma RB proteins family [46, 47], and with BUB1 itself [48] in both HDF_LT/hTERT_ LV AS miR-155 and HDF_LT/hTERT_ LV AS CTRL cells. The inhibition of *miR-155* rescued, at least in part, the effect of SV40 LT on BUB1 function by increasing the protein expression of its targets BUB1, CENP-F, and ZW10.

In conclusion, our results provide a novel direct link between *miR-155* and CIN, which, together with the already described target genes involved in cell cycle control and DNA repair, strengthens the oncogenic features of *miR-155* and highlights its role at the early steps of tumorigenesis.

## MATERIALS AND METHODS

### Cell culture, transfection

HG-3 and MEC-1 cell lines were acquired from Leibniz Institut DSMZ – German Collection of Microorganisms and Cell Cultures in February 2016 and December 2015 respectively. MCF7 and U2OS cell lines were cultured in Dulbecco’s Modified Eagle’s Medium whereas HG-3 and MEC-1 in RPMI, supplemented with 10% fetal bovine serum, 1% Pen/Strep and 1% L-glutamine (Sigma-Aldrich) at 37 °C in a 5% CO_2_ incubator. Human adult dermal fibroblasts (HDFa, Thermo-Fisher Scientific) were cultured in Medium 106 (M106) supplemented with Low Growth Serum Supplement (LSGS) (Thermo-Fisher Scientific). Transient transfections of MCF7, U2OS and HCT116 cell lines were performed using Lipofectamine 2000 (Thermo-Fisher Scientific, Release 14 July 2011) in accordance with the manufacturer’s procedures. Transfection of HG-3 or MEC-1 cells was performed using Amaxa™ Nucleofector™ II (Lonza), program U-016. MicroRNA analysis products were from Thermo-Fisher Scientific (Precursor miR-155, ID:PM12601; antisense inhibitor oligonucleotide, AS miR-155: ID:AM12601 or respective negative controls NC and AS NC). U2OS cells, expressing the YFP-H2B histone were generated using pH2B-YFP expression vector (EUROSCARF).

### RNA extraction and RT-PCR

Total RNA was isolated from cells using QIAzol Lysis Reagent (Qiagen) and treated with DNase (TURBO DNA-free Kit, Thermo Fischer Scientific) to remove contamination of genomic DNA. RNA quantification was performed by NanoDrop 2000 (Thermo-Fisher Scientific). 700 ng of total RNA was reverse-transcribed with the High Capacity cDNA Reverse Transcription Kit (Thermo-Fisher Scientific). Polymerase Chain Reaction (PCR) was performed using AmpliTaq Gold DNA Polymerase (Thermo-Fischer Scientific).

### Plasmids

The 3’UTR region of human *BUB1, ZW10* and *CENP-F* 3′UTRs genes were amplified by PCR from HDF genomic DNA. The amplified fragment was cloned downstream of the Renilla luciferase gene of the psiCHECK-2 luciferase vector (Promega). QuikChange XL Site-Directed Mutagenesis Kit (Agilent technologies) was used for direct mutagenesis.

### Luciferase target assays

U20S cells were co-transfected with psiCHECK-2_BUB1 3’UTR, psiCHECK-2_CENP-F 3’UTR and psiCHECK-2_ZW10 3’UTR (in reference or mutated forms; 150 ng/10,000 cells) and precursor miR-155 or NC (100 nM in the culture medium). After 24 h, firefly and *Renilla* luciferase activities were measured using the Dual-Glo Luciferase Assay System (Promega).

### Reverse transcription quantitative PCR (RT-qPCR)

Assay Design Center software (http://lifescience.roche.com/shop/products/universal-probelibrary-system-assay-design) was used to identify primers and UPL probes (Supplementary Table 2). Total RNA (300 ng) was reverse-transcribed with High Capacity cDNA Reverse Transcription Kit (Thermo-Fisher Scientific). MicroRNAs were reverted from 25 ng of total RNA using the specific reverse primer (stem loop RT primers) and the TaqMan Micro-RNA Reverse Transcription Kit (Thermo-Fisher Scientific). Reactions were incubated 30 min at 16°C, followed by pulsed RT [49]. Real-time PCR was performed using the Universal Mastermix (Roche) and the relative expression, normalized to an endogenous reference *GAPDH* or RNU44, was determined using the 2^−Δct^ method (User Bulletin #2, Applied Biosystems).

### Western blotting

Cell lysates having equal protein concentrations were loaded on a Criterion TGX 4–20% precast polyacrylamide gel (Bio-Rad) and transferred onto a PVDF membrane. Primary antibodies: anti-CENP-F (ab5) (Abcam), anti-BUB1 (B3), anti-ZW10 (3363C4a), anti-vinculin (H-300), anti-γ-tubulin (C-20), anti α-tubulin (Santa Cruz Biotechnology) and anti β-actin (Sigma Aldrich). HRP-conjugated secondary antibody (Cell Signaling Technology) was used. ImageJ (Fiji software, https://fiji.sc/) were used to quantify western blot signals.

### Flow cytometry

Permeabilized cells (70% ethanol, 30 min) were incubated with antibody anti-phospho-histone H3 (Cell Signaling Technology) and with the secondary antibody (10 μg/ml, Alexa Fluor 633 goat anti-rabbit IgG, Thermo-Fisher Scientific) in accordance with the manufacturer’s procedures. Cells were washed and stained with Vybrant DyeCycle Violet Stain (1 μM, Thermo-Fisher Scientific). Analyses were performed using FlowJo v 8.8.6 software.

### Generation of viruses and cell infections

MuLV retroviral particles were obtained using HEK 293T as the packaging cell line and pBABE-puro-SV40LT (gift from Thomas Roberts; Addgene plasmid #13970) [50] and pBabe-hygro-hTERT, pUMVC and pCMV-VSV-G (gifts from Bob Weinberg; Addgene plasmids #1773, #8449 and #8454 respectively) [51, 52] in accordance with the protocol of Scott Dessain and Hua-yin Yu, Weinberg Lab (https://www.addgene.org/static/data/84/58/16578800-af64-11e0-90fe-003048dd6500.pdf). LV pmiRZip AS miR-155, LV pmiRZip CTRL, LV pCDH_miR-155 and LV pCDH_CTRL were obtained by using the pPACKH1 HIV Lentivector Packaging Kit (System Biosciences). HDF_LT/hTERT_ LV AS miR-155 and control (LV AS CTRL) cells were developed by sequential infections with polybrene (5 μg/ml). Briefly, normal HDF were infected at passage P4, with RV LTSV40 for 3 hours, then were washed and infected overnight with LV AS miR-155, or LV AS CTRL. After 7 days of puromycin selection (0.5 μg/ml) (Santa Cruz Biotechnology), at P6, cells were infected with RV hTERT for 3 hours and selected with hygromycin (50 μg/ml) (InvivoGen). Since LV AS miR-155 contains the copGFP reporter gene, GFP-positive cells were sorted using the FACSAriaIII, 100 μm nozzle (BD Biosciences).

### Immunofluorescence

Cells were incubated with the primary antibody in PBS+ (PBS, 5% FBS, 0.02% sodium azide, and 10 mg/ml BSA) overnight at room temperature. Antibodies: anti-BUB1 (ab9000), anti-ZW10 (ab21582), anti γ-tubulin (ab7291), anti-CENP-F (ab5) (Abcam), and human anti-CREST(Antibodies Inc.). Secondary antibodies were from Bethyl Laboratories. DNA was detected with DRAQ5 (Cell Signaling Technology). Coverslip were mounted with SlowFade® Gold antifade reagent (Thermo-Fisher Scientific). Kinetochore tension was indirectly determined by measuring the inter-kinetochore distance of kinetochore pairs on confocal sections when both sister kinetochores were in the same focal plane. To quantify the amount of protein bound to the kinetochore, the pixel intensities of kinetochores from 10-20 nocodazole-treated cells were measured using ImageJ software. To normalize the fluorescent intensity of the protein of interest, anti-centromere staining was performed in the same region. All the fluorescence intensities were acquired with identical settings and corrected by background subtraction using a Carl Zeiss LSM510 META (Zeiss Axiovert 200 and a 63X Plan Neofluar objective).

### Time-lapse experiments

U2OS cells stably expressing YFP-H2B and infected with either LV CTRL or LV miR-155 were seeded into glass-bottomed dishes (Greiner Bio-one) and placed into the incubator of the microscope (system S, Zeiss) set at 37°C in an atmosphere of 5% CO_2_ and 20% humidity. Images were captured every 5 min with a Plan Neofluar oil-immersion objective (40X). The laser intensity was maintained at a minimum to avoid phototoxicity.

### Karyotype analyses

To prepare metaphase spreads, 1–2 × 10^6^ HDFs were seeded (at P14 of two independent experiments) in Amniodishes (Euroclone). Colcemid (Irvine Scientific) treatment (0.5 μg/ml) was carried out after 24 h for 4–5 hours at 37 °C in an atmosphere of 5% CO_2_ and 20% humidity. Cells were fixed in Carnoy’s solution (75% methanol, 25% acetic acid) and stained with Giemsa solution (5% for 10 min). Digital images were analyzed by Genikon software (Nikon).

### 3′Rapid Amplification cDNA Ends (RACE)

Total RNA (2 μg) from HG-3 cells was reverse-transcribed using the oligo-dT adapter primer (AP). The first-strand cDNA was then amplified using HotStarTaq DNA Polymerase (Qiagen) using a 3′ abridged universal amplification primer (AUAP), and a gene-specific reverse primer (BUB1_3UTR). A semi-nested PCR was performed using BUB1_3994 forward primer. The PCR products were cloned into pCR4-TOPO TA vector (Thermo-Fisher Scientific) and sequenced using M13 rev (-29) primer by Sanger method.

### Statistical analyses

Data regarding luciferase assays, times of anaphase onset, RT-qPCR assays, inter-kinetochore distances and kinetochore recruitments were evaluated using two-tailed Student’s t or Mann-Whitney’s tests, based on the significance obtained by the Shapiro-Wilk Normality Test. The significance of differences between percentages of cells blocked at the G2/M phase was assessed using the one sample *t* test (two tailed, with theoretical median value = 1.0) after Kolmogorov-Smirnov test with Dallal-Wilkinson-Lillie correction for *p* value normality. In this experiment, we applied Kolmogorov-Smirnov test because of the small set size. The two-tailed Fisher’s exact test was used for metaphase plate morphology and karyotype analyses. All RT-qPCR assays were performed in triplicate. The number of analyzed samples is shown in the Figures (n), a *p*-value > 0.05 was considered not significant (ns), significant *p*-values are indicated in the figures. When indicated, standard deviation (SD) is represented as a scale bar on graphs.

## SUPPLEMENTARY MATERIALS FIGURES AND TABLES








