# Supplementary material for: Hsa-miR-155-5p drives aneuploidy at early stages of cellular transformation
Source: Oncotarget. 2018 Feb 7;9(16):13036–47. doi: 10.18632/oncotarget.24437 (PMC5849193; doi:10.18632/oncotarget.24437)
Supplement: Supplementary file 1 [file oncotarget-09-13036-s001.pdf]

Hsa-miR-155-5p drives aneuploidy at early stages of cellular transformation

SUPPLEMENTARY MATERIALS

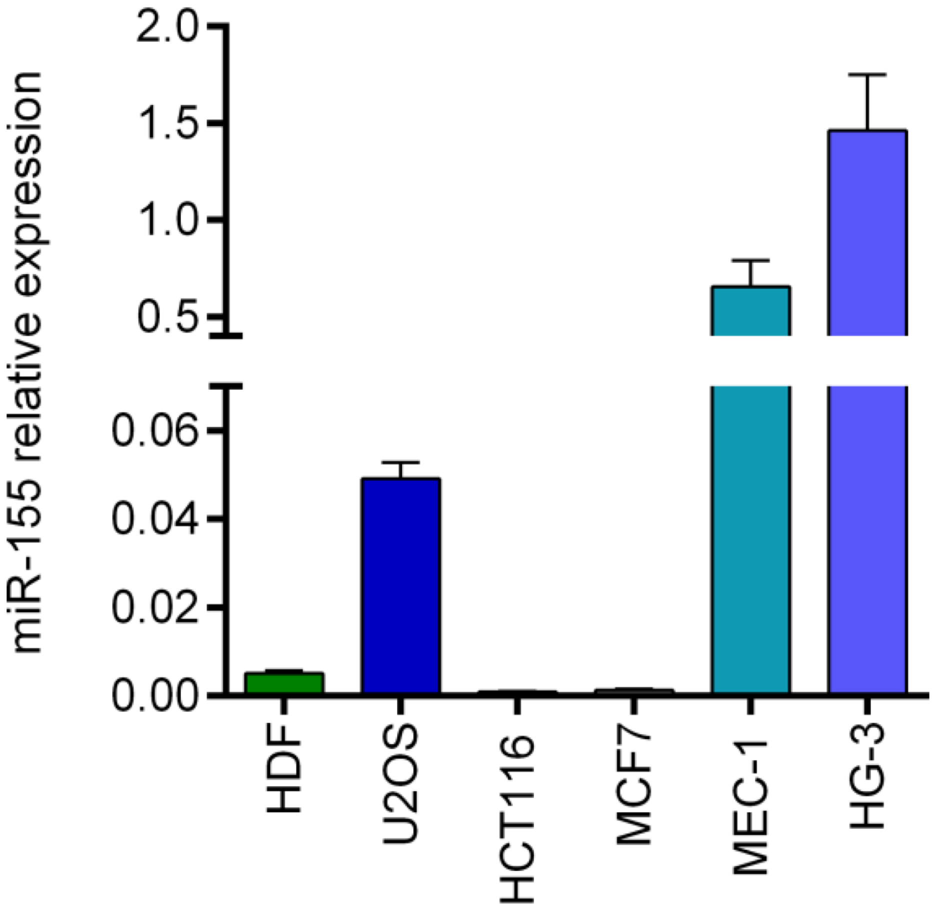

Supplementary Figure 1: *miR-155* relative expression in different cell lines. Relative expression was determined using RT-qPCR. Each sample was normalized to the endogenous reference RNU44 ($2^{-\Delta ct}$ method).

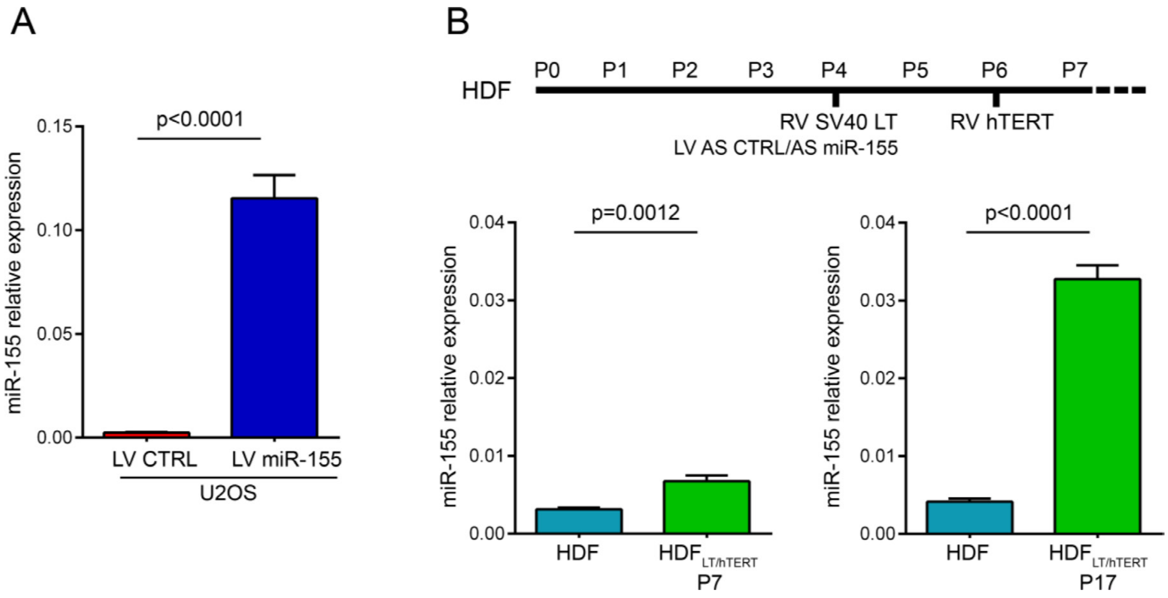

Supplementary Figure 2: *miR-155* relative expression in U2OS, HDF and $HDF_{LT/hTERT}$ cells at different passages (P7, P17). (**A**) U2OS cells were infected either with a lentivirus encoding *miR-155* (LV miR-155) or control (LV CTRL). (**B**) infection timeline for the *in vitro* transformation of HDF by ectopic expression of SV40 Large T antigen (RV SV40 LT), and human telomerase (RV hTERT) (upper panel) and for inhibition of miR-155 expression by lentivirus encoding antisense miR-155 (LV AS miR-155) or control (LV AS CTRL). *miR-155* relative expression in normal HDF and at different passages of $HDF_{LT/hTERT}$ cells (bottom panel). Relative expression was determined using RT-qPCR. Each sample was normalized to the endogenous reference RNU44 ($2^{-\Delta ct}$ method).

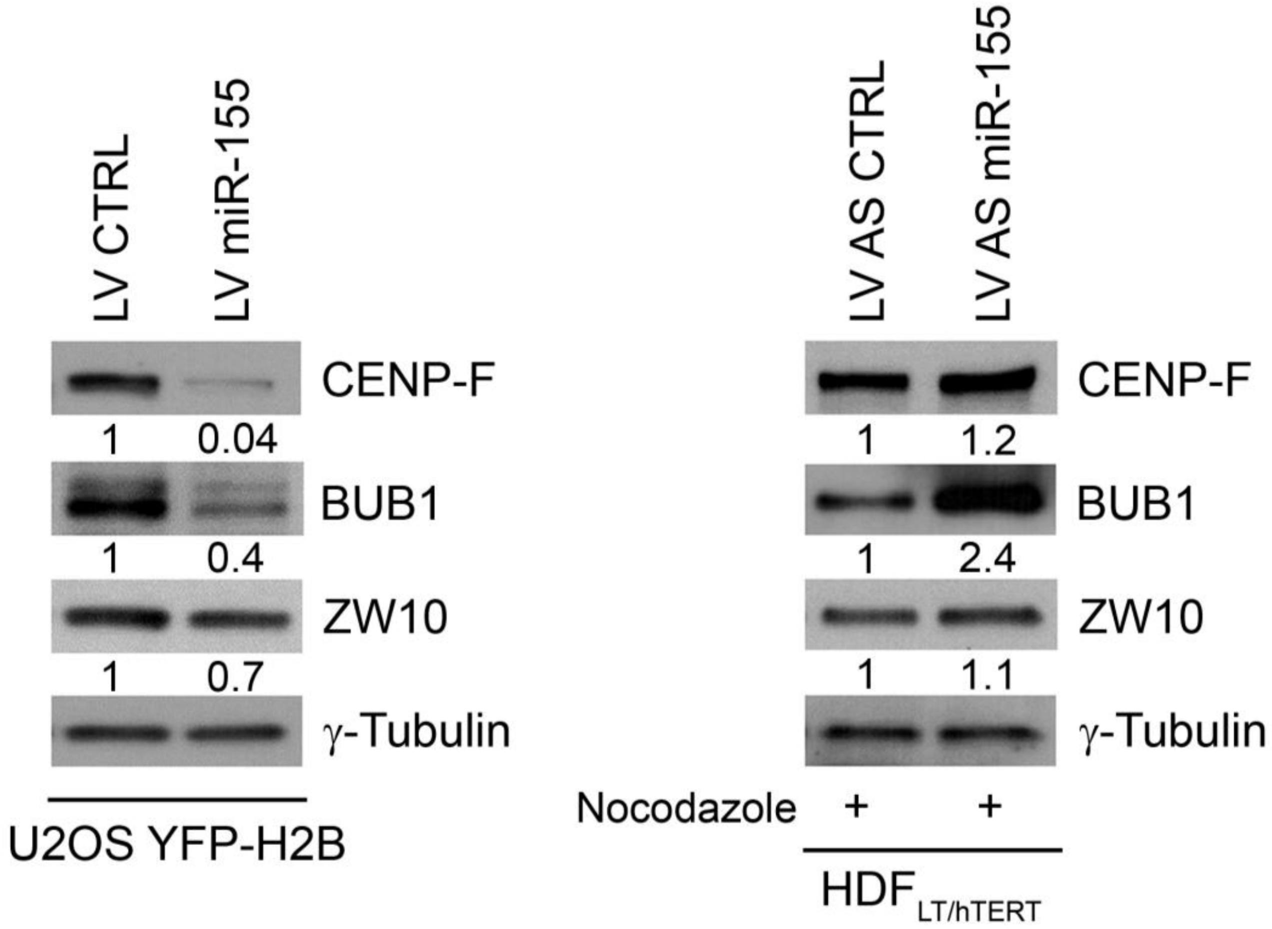

Supplementary Figure 3: *miR-155* regulates CENP-F, BUB1 and ZW10. Western blots of lysates of U2OS YFP-H2B cells (left) and $HDF_{LT/hTERT}$ (right), infected with lentiviruses (LV) as indicated. $HDF_{LT/hTERT}$ cells were blocked at early M-phase with nocodazole (75 ng/ml; 17 h). γ-Tubulin was used as loading control. Results of densitometric analysis are shown.

Supplementary Table 1: Karyotype of $HDF_{LT/hTERT}$ cells at passages 14. See_Supplementary_Table 1

Supplementary Table 2: Oligonucleotide primers used in the study for cloning, gene expression (GE), microRNA expression (miRE), reverse transcription (RT), 3′ rapid amplification cDNA ends (RACE), mutagenesis, and gel electrophoresis (Gel elect.)

Name	Forward primer	Reverse primer	Probe/Assay	Method
CENPF_3′UTR	CCG**CTCGAG**GGCTGTGCCTACAGGACTTC	ATAAGAAT**GCGGCCG**CCCCGA TACAAAACTATTCCATGCC		Cloning
ZW10_3′UTR	CCG**CTCGA**GATCGCCCATTGGTTTTGGTG	ATAAGAAT**GCGGCCG**CGTGGCCCA TTCCTCCCTTC		Cloning
BUB1_3′UTR	CAA**TCTAGA**TGAATCTGCTCACTTTA AACCTGT	CAA**TCTAGAG**CGACTGTTGAAAAG CATGA		Cloning
CENPF_3′UTR_MUT	GATCACCTGTGCCGGCCATTCCTCTA CTGCAATG	GTAGAGGAATGGCCGGCACAGGTG ATCAGTGCTTCC		Mut.
ZW10_3′UTR_MUT	CAAACTGTCAGGCGCCGATTT CATGCTGCTTCCAGAG	GCAGCATGAAACGGCGCCTGA CAGTTTGTTAGCTAATC		Mut.
BUB1_3′UTR_MUT	TCCCTTGTTCCCGGGTTCGAATGAA AACAAATCCCAGGTTATGCAATCC	ATTTGTTTTCATTCGAACCCGG GAACAAGG GAAGGAGGGATGG		Mut.
BUB1_3328-4090	CTGATTGGGCTGCTTCTGG	GAACTGTGCATAACCTGGGA		Gel
BUB1_4090		GAACTGTGCATAACCTGGGA		Gel elect.
BUB1_3569-4090	CTGCTCTTAGAATGTAAGCG	GAACTGTGCATAACCTGGGA		Gel elect.
155 F	GCGGCGGTTAATGCTAATCGTG	GTGCAGGGTCCGAGGT	UPL21/Fam-Q	miRE
RT_155	GTTGGCTCTGGTGCAGGGTCCGAG GTATTCGCACCAGAGCCAACACCCCT			RT
U44_F	GCGGCGGCCTGGATGATGATAG	GTGCAGGGTCCGAGGT	UPL21/Fam-Q	miRE
RT_U44	GTTGGCTCTGGTGCAGGGTCCGAGG TATTCGCACCAGAGCCAACAGTCAGTT			RT
U19_hTERT	GCCTTCAAGAGCCACGTC	CCACGAACTGTCGCATGT	UPL19/Fam-Q	GE
GAPDH		LIFETECH REF:4333764T		GE
SV40 LT-F	GCGGCGGACCUGGCAUACAAU	GACTCAGGGCATGAAACAGG	SYBR	GE
BUB1_3994	GGGCCTTCTAGCCTGGAC			RACE
AP		GGCCACGCGTCGACTAGTACTTTT TTTTTTTTTTTT		RACE
AUAP		GGCCACGCGTCGACTAGTAC		RACE

The sequences of the UPLs-Locked Nucleic Acids (Universal Probe library, Roche) used for qPCR are indicated. Fam is the 6-carboxyfluorescein dye and Q is the fluorescein quencher. Nucleotides in bold type represent the restriction enzyme sequence added to the specific oligonucleotide for vector cloning.

Supplementary Movie 1: MO1 - U2OS LV CTRL. See_Supplementary_Movie 1

Supplementary Movie 2: M02 - U2OS LV miR-155. See_Supplementary_Movie 2
