# Supplementary material for: Hsa-miR-155-5p drives aneuploidy at early stages of cellular transformation
Source: Oncotarget. 2018 Feb 7;9(16):13036–47. doi: 10.18632/oncotarget.24437 (PMC5849193; doi:10.18632/oncotarget.24437)
Supplement: Supplementary file 2 [file oncotarget-09-13036-s002.docx]

| **Supplementary Table 1. Karyotype of HDF_LT/hTERT_ cells at passages 14** | | | | |
| --- | --- | --- | --- | --- |
| **Metaphases** | ***Experiment 1*** | | ***Experiment 2*** | |
|  | ***LV AS CTRL*** | ***LV AS miR-155*** | ***LV AS CTRL*** | ***LV AS miR-155*** |
| 1 | 45, XX, -13 | 46, XX | 46, XX | 46, XX, del(11q) |
| 2 | 44, XX, -8, -22 | 47, XX, +mar | 46, XX, del(9q) | 46, XX |
| 3 | 46, XX, -10, +6 | 46, XX | 46, XX | 46, XX |
| 4 | 46, XX, -22, +7, del(Xq) | 46, XX | 45, XX, -8, -11, +7 | 46, XX |
| 5 | 46, XX, del(Xq) | 46, XX, del(10p) | 46, XX | 45, XX, -9, -15, -22, +2mar |
| 6 | 45, XX, del(9p) | 45, XX, -7 | 46, XX | 46, XX |
| 7 | 46, XX | 46, XX | 46, XX | 46, XX |
| 8 | 46, XX, der(13), del(19p) | 46, XX | 45, XX, -16 | 46, XX |
| 9 | 46, XX, del(8p) | 44, XX, -8, -10, -19, -22, +3mar | 45, XX, -16, del(1q) | 46, XX |
| 10 | 90 | 45, XX, -10 | 46, XX, del(11p) | 47, XX, del(4q), del(7p), +1, der(17) |
| 11 | 46, XX | 43, X, -9, -14, -21, -X, +mar | 45, XX, -13 | 46, XX |
| 12 | 45, XX, -22, del(2p), t(2;22) | 44, XX, -13, .17, -18, +mar | 46, XX | 46, XX, del(10q) |
| 13 | 45, XX, -15, -5, +mar | 46, XX | 46, XX | 46, XX |
| 14 | 46, XX | 43, X, -X, -8, -19 | 45, XX, -8, -18, +14 | 46, XX |
| 15 | 46, XX, del(9p), del(20p) | 46, XX | 46, XX | 46, XX, del(2q) |
| 16 | 44, XX, -15, -22, del(7p), der(13) | 45, XX, -2, -21, der(22) | 44, XX, -6, -8, -19, del(5q), +mar | 46, XX |
| 17 | 40, XX, -11, -14, -15, -19, -20, -22 | 46, XX | 46, XX | 46, XX, del(3q), del(5p) |
| 18 | 46, XX | 46, XX | 46, XX | 46, XX |
| 19 | 46, XX | 46, XX | 47, XX, +14, del(15q), -22, +mar | 46, XX |
| 20 | 46, XX, der(18) | 46, XX | 46, XX | 46, XX, del(10p) |
| 21 | 46, XX | 46, XX | 46, XX | 46, XX |
| 22 | 46, XX, del(Xq) | 46, XX | 46, XX, del8p, der(X) | 46, XX |
| 23 | 44, XX, -8, -22 | 46, XX, del(Xq) | 46, XX | 46, XX, del(Xq) |
| 24 | 46, XX | 46, XX, del(Xq) | 46, XX | 46, XX |
| 25 | 46, XX, del(8p) | 46, XXX, -7, del(8p) | 45, XX, -8 | 47, X, -X, +9, +mar |
| 26 | 46, XX, del(20p) | 46, XX, del(9p) | 45, XX, -6, -19, +mar, del(20q) | 46, XX |
| 27 | 46, XX, -16, +22 | 46, XX, -6, -14, +9, +11, del(4p) | 46, XX | 46, XX |
| 28 | 46, XX | 46, XX | 46, XX | 46, XX |
| 29 | 46, XX | 46, XX | 46, X, -X, -8, der(3), +mar | 46, XX |
| 30 | 45, XX, -13, del(12q) | 46, XX, -4, +mar, del(3p) | 46, XX | 46, XX |
| 31 | 46, XX, | 45, XX, -2, -5, +11 | 46, XX | 46, XX |
| 32 | 46, XX, -6, der(6), t(3;6) | 46, XX, del(4p) | 46, XX | 46, XX |
| 33 | 46, XX | 46, XX | 46, X, -X, del(1p), add(20), +4, -6 | 46, XX |
| 34 | 45, X, -X, der(13), der(8) | 46, XX | 46, XX | 46, XX, del(10p) |
| 35 | 46, XX | 46, XX, del(2p), del(7q) | 46, XX, add(3) | 46, XX |
| 36 | 46, XX | 46, XX, add(17) | 46, XX | 46, XX |
| 37 | 45, X, -X, del(5q) | 46, XX | 46, XX | 46, XX |
| 38 | 46, XX | 46, XX | 46, XX, -15, +13 | 46, XX, del(5q) |
| 39 | 45, XX, -11, t(14;17) | 46, XX | 46, XX, add(11) | 46, XX, del(8q), -21, +17 |
| 40 | 46, XX | 44, XX, -15, -19 | 45, XX, -22 | 46, X, -X, +mar |
| 41 | 46, XX | 46, XX | 45, XX, -22 | 46, XX |
| 42 | 91 | 46, XX, -4, +mar | 46, XX, add(2) | 46, XX |
| 43 | 46, XX | 46, XX | 46, XX | 46, XX, del(Xq) |
| 44 | 46, XX | 46, XX, del(6q) | 46, XX | 46, XX |
| 45 | 90 | 45, XX, -19, del(1p) | 46, XX | 46, XX |
| 46 | 47, XX, del(Xq), +7 | 47, XX, +mar, del(16q) | 46, XX, del(7q) | 46, XX |
| 47 | 47, XX, +7 | 46, XX | 46, XX, +mar, del(9q) | 46, XX |
| 48 | 46, XX | 44, XX, -16, -19, del(5p) | 46, XX | 46, XX |
| 49 | 45, XX, -8, del(9q), del(2p) | 46, XX, +6, -12, del(19) | 46, XX | 46, XX |
| 50 | 43, XX, -15, -18, -22 | 46, XX | 46, XX, del(2p) | 46, XX |
| 51 | 45, XX, -22 | 46, XX, del(2q) | 46, XX | 46, XX |
| 52 | 44, XX, -8, -13 | 45, XX, -21, del(1q), add(17) | 46, XX | 46, XX |
| 53 | 45, XX, -4q, -17, +20 | 46, XX | 45, XX, -18 | 46, XX |
| 54 | 46, XX | 46, XX, del(6q) | 46, XX | 46, XX, -20, +22, t(20;21) |
| 55 | 44, XX, -9, -20 | 46, XX | 46, XX | 46, XX |
| 56 | 45, XX, -17 | 46, XX | 46, XX, -15, +mar | 46, XX |
| 57 | 44, XX, -11, -14 | 45, XX, -14 | 44, XX, -21, -22 | 46, XX |
| 58 | 45, XX, -6 | 46, XX | 46, XX | 46, XX |
| 59 | 47, XX, -6, -8, +3mar | 47, XX, +mar | 45, XX, -12 | 46, XX, -21, +mar, del(10p) |
| 60 | 46, XX | 45, X, -2, -X, +mar | 46, XX, +6, -10 | 46, XX |
| 61 | 46, XX, del(9q) | 46, XX | 46, XX, -1, -18, +2mar | 46, XX, del(6p) |
| 62 | 45, XX, -8, -9, del(Xq), +mar | 46, XX | 46, XX, del(4q) | 46, XX |
| 63 | 47, XX, -8, -9 , +3mar | 46, XX, -14, +20 | 46, XX | 46, XX |
| 64 | 47, XX, del(4p), +mar | 45, XX, del(4q), -7, add(10), +11q | 45, XX, -22 | 46, XX |
| 65 | 47, XX, +mar, del(11p) | 46, XX | 46, XX | 46, XX |
| 66 | 45, XX, -13, t(10;13) | 45, X, -X, -2, +11, -8, +mar | 46, X, -8, -X, +10, +mar | 46, XX |
| 67 | 46, XX, -22, +15, del(10q) | 46, XX, del(2q), add(9) | 45, XX, -4 | 46, XX |
| 68 | 46, XX, add(14) | 46, XX, del(Xq), del(7q), -17, -18, +3mar | 46, XX | 46, XX |
| 69 | 43, XX, -4, -10, -12, -13, -14, +2mar | 46, XX | 45, XX, -15 | 46, XX, del(2p) |
| 70 | 45, XX, -10 | 46, XX | 46, XX, del(3p) | 45, X, -X |
| 71 | 46, XX | 45, XX, -13 | 45, XX, -22 | 46, XX |
| 72 | 45, XX, -22 | 47, XXX, del(6q) | 46, XX | 46, XX |
| 73 | 46, XX | 46, XX | 44, XX, -8, -22 | 46, XX |
| 74 | 46, XX, der(17), -2, +mar | 46, XX, del(20p) | 45, XX, -19, t(11;19) | 46, XX, -7, +mar |
| 75 | 45, XX, -4 | 46, XX | 46, XX | 46, XX |
| 76 | 45, XX, -15, del(10p) | 46, XX | 46, XX | 45, XX, -4, +7, -8, +mar |
| 77 | 46, XX | 46, XX, del(4p) | 94 | 46, XX |
| 78 | 46, XX | 45, XX, -18 | 46, XX | 46, XX, del(9q), -21, +mar |
| 79 | 46, XX | 46, XX, -20, +mar, del(2q) | 46, XX | 46, XX |
| 80 | 45, XX, -10, -20, +mar | 44, X, -X, -22, -12, +add(14) | 46, XX | 46, XX |
| 81 | 46, XX | 46, XX, del(19) | 46, XX | 46, XX, +7, -8, +14, -15, der(17) |
| 82 | 46, XX | 46, XX, -8, +7, +10 | 46, XX | 47, XX, -8, +mar |
| 83 | 45, XX, -1, -15, -18, -22, +3mar, der(21) | 46, XX, del(10p) | 44, XX, -15, -22, add(7) | 46, XX |
| 84 | 46, XX, -18, +mar | 46, XX, del(9p) | 46, XX | 46, XX |
| 85 | 46, X, +mar | 46, XX, -10, +mar | 46, XX | 46, XX |
| 86 | 44, XX, -2, -6, -22, +mar | 46, XX | 46, XX | 46, XX |
| 87 | 46, XX | 46, XX | 46, XX | 46, XX |
| 88 | 46, XX | 45, XX, -5, der(17), del(20q) | 46, XX | 46, XX |
| 89 | 46, XX | 46, XX | 46, XX, del(6q) | 46, XX, del(16q) |
| 90 | 46, XX, -21, +19, del/7p), add(9) | 45, XX, -18 | 45, XX, -22 | 45, X, -X |
| 91 | 45, XX, -2, del(16q) | 45, XX, -7, add(X) | 46, XX | 46, XX |
| 92 | 45, XX, -8 | 46, XX | 46, XX | 46, XX |
| 93 | 46, XX, +7, -10 | 46, XX | 46, XX, del(7p) | 46, XX |
| 94 | 46, XX | 46, XX | 45, XX, -21 | 46, XX, del(12p) |
| 95 | 45, XX, -17 | 46, XX | 46, XX, -9, +mar | 46, XX |
| 96 | 45, XX, -10 | 46, XX | 46, XX, del(4p), add(11) | 45, XX, -4 |
| 97 | 47, XX, +7, -21, +mar | 46, XX, -20, +mar | 46, XX | 46, XX, del(11p) |
| 98 | 47, XX, +7, del(Xq), del(5p) | 46, XX, -11, -16, +22, del(6q), +mar | 46, XX, del(20q) | 46, XX |
| 99 | 46, XX, +7, -6 | 45, XX, -14, -20, +mar, del(Xq) | 45, XX, -22 | 46, XX |
| 100 | 42, XX, -10, -16, -20, -21 | 46, XX, -18, +mar | 45, XX, -22 | 46, XX |
| 101 | 46, XX | 46, XX | 46, XX | 46, XX |
| 102 | 46, XX, -4, +mar | 46, XX, add(5), -6, +mar | 46, XX | 46, XX, del(1q), add(9) |
| 103 | 46, XX, +7, -5 | 46, XX, del(18q) | 45, XX, -21 | 46, XX |
| 104 | 45, XX, -7 | 46, XX, del(11q) | 45, X, -X | 46, XX |
| 105 | 45, XX, -2, -12, +7 | 46, XX | 46, XX | 46, XX |
| 106 | 46, XX | 46, XX | 46, XX | 46, XX |
| 107 | 46, XX, del(Xp) | 45, XX, -16 | 45, XX, -22 |  |
| 108 | 48, XX, +7, +mar, del(12p) | 46, XX | 47, XX, +8 |  |
| 109 |  | 46, XX | 46, XX |  |
| 110 |  | 45, XX, -13 | 46, XX, -14, -12, del(9q) |  |
| 111 |  | 46, XX |  |  |
| 112 |  | 46, XX |  |  |
| 113 |  | 46, XX |  |  |
| 114 |  | 46, XX, +20, -7 |  |  |
| 115 |  | 46, XX |  |  |
